# Rotating Night Shift Work, Sleep, and Thyroid Cancer Risk in the Nurses’ Health Study 2

**DOI:** 10.3390/cancers15235673

**Published:** 2023-11-30

**Authors:** Kyriaki Papantoniou, Peter Konrad, Shahab Haghayegh, Susanne Strohmaier, A. Heather Eliassen, Eva Schernhammer

**Affiliations:** 1 Department of Epidemiology, Center for Public Health, Medical University of Vienna, 1090 Vienna, Austria; peter.konrad@meduniwien.ac.at (P.K.); susanne.strohmaier@meduniwien.ac.at (S.S.); eva.schernhammer@muv.ac.at (E.S.); 2 Channing Division of Network Medicine, Brigham and Women’s Hospital and Department of Medicine, Harvard Medical School, Boston, MA 02115, USA; shaghayegh@bwh.harvard.edu (S.H.); heliasse@hsph.harvard.edu (A.H.E.); 3 Department of Epidemiology, Harvard T.H. Chan School of Public Health, Boston, MA 02115, USA; 4 Department of Nutrition, Harvard T.H. Chan School of Public Health, Boston, MA 02115, USA

**Keywords:** night shift work, sleep duration, sleep difficulty, thyroid cancer

## Abstract

**Simple Summary:**

This study investigated the potential connection between working night shifts, sleep patterns, and the risk of thyroid cancer among nurses. The researchers followed over 114,000 women for 26 years and identified 588 cases of thyroid cancer. The study found that there was no link between working night shifts and thyroid cancer risk. However, it did suggest a possible association between frequently having difficulty falling or staying asleep and a higher risk of thyroid cancer. Among nurses who worked night shifts for over 10 years and experienced sleep difficulties most of the time, there was a modestly increased risk of thyroid cancer. In summary, the research suggests that sleep problems among those who work night shifts may be linked to an increased risk of thyroid cancer. However, more research is needed to confirm these findings and better understand the relationship between night shift work, sleep patterns, and thyroid cancer.

**Abstract:**

Night shift work has been associated with breast, prostate, and colorectal cancer, but evidence on other types of cancer is limited. We prospectively evaluated the association of rotating night shift work, sleep duration, and sleep difficulty with thyroid cancer risk in the Nurses’ Health Study 2 (NHS2). We assessed rotating night shift work duration (years) at baseline and throughout follow-up (1989–2015) and sleep characteristics in 2001. Cox proportional hazard models, adjusted for potential confounders, were used to calculate hazard ratios (HR) and 95% confidence intervals (CI) for (a) shift work duration, (b) sleep duration, and (c) difficulty falling or staying asleep. We stratified the analyses of night shift work by sleep duration and sleep difficulty. Over 26 years of follow-up, 588 incident cases were identified among 114,534 women in the NHS2 cohort. We observed no association between night shift work and the risk of thyroid cancer. Difficulty falling or staying asleep was suggestively associated with a higher incidence of thyroid cancer when reported sometimes (HR 1.26, 95% CI 0.95, 1.66) and all or most of the time (HR 1.35, 95% CI 1.00, 1.81). Night shift workers (10+ years) with sleep difficulty all or most of the time (HR 1.47; 0.58–3.73) or with >7 h of sleep duration (HR 2.17; 95% CI, 1.21–3.92) had a higher risk of thyroid cancer. We found modest evidence for an increased risk of thyroid cancer in relation to sleep difficulty, which was more pronounced among night shift workers.

## 1. Introduction

Thyroid cancer is the most common endocrine malignancy (fifth most common female cancer), with a sharply rising incidence in both sexes and especially among younger women [1,2]. The increase in thyroid cancer rates and, in particular, those of papillary thyroid cancer—the most common histological subtype—could be largely explained by the introduction of new diagnostic techniques and increased medical surveillance [3,4]. However, overdiagnosis only partly accounts for the increase in thyroid cancer incidence, and other modifiable risk factors likely play a role [5]. Changes in patterns of exposure to traditional risk factors (e.g., age, sex, obesity, family history, diagnostic ionizing radiation exposure, endocrine chemicals) for thyroid cancer may also contribute to the upward trend [6]. New or emerging environmental and occupational exposures, such as night shift work and the increasing use of light at night, can lead to circadian disruption and sleep disturbances that have been previously associated with hormone-related cancers and may play a role in the etiology of thyroid cancer [7].

Shift work is one of the most prevalent occupational exposures, with an estimated 16.4% of workers engaged in a non-daytime work schedule in 2017–2018 in the United States [8]. Night shift work has been classified by the International Agency for Research on Cancer (IARC) of the World Health Organization as a probable (class 2A) human carcinogen for breast, prostate, and colorectal cancer [9]. Furthermore, one out of four shift workers suffers from shift work disorder [10]—defined as clinically significant insomnia symptoms or sleepiness that leads to impairment of daytime functioning [11]. It has been hypothesized that co-exposure to night shift work and sleep disturbances may lead to an increased risk of chronic diseases, including gastrointestinal cancer [12], but data on other tumors are scarce.

The circadian clock and sleep regulate the hypothalamus–pituitary–thyroid axis and thyroid function, including the daily production and secretion of thyroid hormones [13]. Sleep disturbances have been associated with an increase in thyroid cancer risk [14,15]. Several studies suggest a link between night work, benign thyroid disease, and thyroid hormone disturbances [16,17], including higher thyroid stimulating hormone (TSH) levels [18], a marker linked to thyroid cancer risk [19,20]. However, to date, no previous study has evaluated the association between night shift work and thyroid cancer risk in humans.

In this prospective study, we evaluated the association of night shift work and sleep disturbances with incident thyroid cancer risk in female nurses. We further assessed the potential joint effects of night shift work and disturbed sleep on thyroid cancer risk. We hypothesized that women with long-term night shift work and/or frequently disturbed sleep would be at an increased risk of thyroid cancer compared to women without night shift work and/or sleep disturbances. 

## 2. Materials and Methods:

### 2.1. Study Design

The Nurses’ Health Study 2 (NHS2) is an ongoing prospective cohort study established in 1989 with an enrollment of 116,429 US registered female nurses aged 25–42 years. Participants returned a mailed questionnaire with detailed information at baseline in 1989 and updated the information biennially thereafter. The NHS2 obtained ethical approval from the Institutional Review Board of Brigham and Women’s Hospital (Boston, Massachusetts) and the Harvard T. H. Chan School of Public Health (Protocol #: 1999P003389). Informed patient/participant consent was obtained from study participants. The research was completed in accordance with the Declaration of Helsinki, as revised in 2013.

### 2.2. Assessment of Rotating Night Shift Work

NHS2 participants were asked about their rotating shift work history in 1989 through the following question: “What is the total number of years during which you worked rotating night shifts (at least 3 nights/month in addition to days/evenings in that month)?” and the following pre-specified categories: 1–2 years, 3–5 years, 6–9 years, 10–14 years, 15–19 years, 20+ years. Subsequently, the total number of months having worked rotating night shifts in the years since the last assessment was assessed in 1991, 1993, 1997, 2001, and 2005. The follow-up estimates were added to the baseline assessment to calculate the updated total cumulative duration (in years) of rotating night shift work history.

### 2.3. Assessment of Sleep Duration and Sleep Difficulty

A subset of participating women (N = 82,786) reported their average number of hours of sleep over a 24-h period on the 2001 questionnaire; response categories were <5, 5, 6, 7, 8, 9, 10+ h. A prior NHS study had found that a single questionnaire-based report of sleep duration correlated highly with sleep duration recorded in daily diaries (r = 0.79) [21]. We collapsed these categories based on the sample distribution by grouping women into categories of ≤5, 6, 7, 8, or ≥9 h of sleep per 24-h period. In 2001, the nurses recorded how often, during the past 4 weeks, they had difficulty falling asleep or staying asleep. The possible responses were: “all of the time”, “most of the time”, “a good bit of the time”, “some of the time”, “little of the time”, and “none of the time”. In the current study, we reclassified participants into 3 categories as follows: “All, most, or a good bit of the time”, “Some of the time”, and “Little or none of the time”.

### 2.4. Thyroid Cancer Assessment

Incidents of thyroid cancer between 1 June 1989 and 31 May 2015 were considered in the shift work analyses, and incident cases between 1 June 2001 and 31 May 2015 in the sleep analyses. Self-reported diagnoses of thyroid cancer were obtained on biennial questionnaires. Participants who reported a diagnosis of cancer were asked for permission to acquire their medical records and pathological reports. A study physician, blinded to shift work and sleep information, reviewed records to confirm the thyroid cancer diagnoses and to extract relevant information such as histology, tumor size, and surgery type.

### 2.5. Covariates

Covariates extracted from the baseline questionnaire included age (months), height (inches), race, age of menarche, BMI at age 18 (kg/m^2^), and alcohol consumption at age 18 (g/day). Time-varying covariates included age, BMI, smoking status, pack-years of cigarettes among smokers (packs/years), physical activity level (MET-hours/week), oral contraceptive use (never, past, current), parity and age at first birth, breastfeeding duration (months), menopausal status, age at menopause, reproductive years, and hormone therapy [22,23]. On the 1989 baseline questionnaire, participants were asked whether they had a history of thyroid disease without specifying hypothyroidism or hyperthyroidism and the date of diagnosis. They were also asked about the use of thyroid hormone replacement therapy. During the follow-up, the use of thyroid hormone replacement therapy, physician diagnoses of hypothyroidism and hyperthyroidism, thyroid nodules, and other hyperthyroidism were queried biennially. From 1993 to 2013, participants were asked about newly diagnosed hypothyroidism or Graves’ disease and the dates of diagnosis for each condition.

### 2.6. Statistical Analyses

Descriptive statistics for covariates were calculated by categories of rotating night shift work history assessed in 1989 and by categories of sleep duration assessed in 2001 using means and standard division for continuous variables and proportions for categorical variables. Cox proportional hazard models were used to evaluate the association of (i) updated rotating night shift work history in years (None (reference), 0.1–1.9, 2–3.9, 4–5.9, 6–7.9, 8–9.9, or 10+ years) and collapsed (None (ref), 1–4.9, 5–9.9, or 10+ years), (ii) average hours of sleep duration (≤5, 6, 7(reference), 8, ≥9 h), and (iii) difficulty falling or staying asleep (little or none of the time (reference), sometimes, all or most of the time) with thyroid cancer risk. Women contributed person-time to the analyses from the return date of the 1989 questionnaire (2001 questionnaire for sleep analyses) until the date of diagnosis, date of death, date of drop-out, date of self-reported cancer other than non-melanoma skin cancer, or until the return of the 2015 questionnaire, whichever came first. Age and multivariable-adjusted hazard ratios (HR) and 95% confidence intervals of incident thyroid cancer were calculated. In multivariable analyses, we adjusted for the following confounders that were a priori selected based on known or suspected risk factors for thyroid cancer and hormonal and reproductive factors that have been previously associated with thyroid cancer incidence in the NHS2 cohort [22]: race, BMI, physical activity, menopausal status, menopausal hormone therapy, duration of menopausal hormone therapy, alcohol consumption, smoking status, parity, age at first birth, reproductive years, i.e., time from menarche to natural menopause, and breastfeeding. In separate models, we additionally mutually adjusted shift work analyses for sleep. Last, we also adjusted analyses for personal history of benign thyroid disease (e.g., hypo- or hyperthyroidism). We performed stratified analyses by categories of sleep duration and difficulty falling or staying asleep, chronotype (morningness or eveningness), BMI (<30, ≥30), and menopausal status (premenopausal, postmenopausal). Finally, sensitivity analyses were performed (a) restricting analyses to papillary tumors (90% of total) and to smaller tumors (<2 cm), (b) including only nurses who answered all shiftwork questionnaires (N = 19,005; 326 cases), and (c) restricting to cancer cases confirmed by medical record only (N = 494 cases). All statistical analyses were performed by SAS (version 9.1; SAS Institute Inc., Cary, NC, USA).

## 3. Results

Compared to day workers, those with 10 or more years of rotating night shift work were slightly older, had higher BMI, had a spouse with lower education, were more likely to be current and heavier smokers, nulliparous, less likely to have breastfed longer than 12 months, and to report the use of postmenopausal hormones and oral contraceptives. Long-term night shift workers were also more likely to report having short sleep (≤6 h) and difficulty falling or staying asleep. Compared to participants with 7 h of sleep, those with short (≤5 h) and long (≥9 h) were slightly older, heavier at baseline, and were current smokers (Table 1).

We observed no overall association between the duration of rotating night shift work and thyroid cancer risk in the age-adjusted and multivariable-adjusted models (<5 years: HR 1.19, 95%CI 0.98, 1.44; 5–9.9 years: HR 1.13, 95%CI 0.86, 1.48, 10+ years HR 1.10, 95%CI 0.79, 1.54) (Table 2). We found evidence for minimal to zero confounding by included covariates in the examined associations of shift work and sleep with thyroid cancer. Additional adjustments for sleep duration and difficulty falling or staying asleep did not materially change the risk estimates. Difficulty falling or staying asleep was borderline associated with thyroid cancer risk when reported sometimes (HR 1.26, 95% CI 0.95, 1.67) and all or most of the time (HR 1.35, 95%CI 1.00, 1.81) (Table 3). No clear association pattern was observed for sleep duration and thyroid cancer risk. Additional adjustments for benign thyroid disease did not change the risk estimates. The association of night work and thyroid cancer in the subsample of participants with available sleep information was similar to the main analysis (Table 4). Among those who reported long sleep (≥7 h), ≥10 years of rotating night shift work was associated with a more than 2-fold increase in the risk of thyroid cancer (HR 2.17, 95%CI 1.21, 3.92), compared to never shift work, though the trend across years was not significant (*p* = 0.39). Shift workers with short sleep also tended to have increased risk for thyroid cancer, especially among workers with shorter shift work experience (0.1–4.9 yrs: 1.39; 0.90, 2.14, 1.58; 0.93, 2.67), but not among those with longer shift work history (10+ yrs: 1.03; 0.53, 2.01) Similarly, night shift workers who reported difficulty falling or staying asleep most or all of the time had increased risks for thyroid cancer across all 3 categories of shift work history (significant only for ≤5 years), whereas no association was found among those reporting some or little/no sleep difficulty, though the interaction was not statistically significant (*p* = 23). In secondary analyses of night shift work, we found no evidence of effect modification by obesity, menopausal status, or chronotype (Supplementary Table S1). Effects of sleep duration and sleep difficulty were slightly stronger among non-obese, premenopausal women and participants with a morningness chronotype, although the interaction tests were not statistically significant. (Supplementary Tables S2 and S3). Participants with short sleep duration (<7 h) and difficulty falling or staying asleep all or most of the time had an increased risk of thyroid cancer (Supplementary Table S3). In sensitivity analyses, risk estimates were unchanged when restricting the analysis to papillary thyroid cancer and somewhat stronger for small (<2 cm) tumors (Supplementary Table S4). Results were similar in direction and magnitude to the main analyses when we restricted our sample to participants who answered all shift work questionnaires (Supplementary Table S5) and to thyroid cancer cases that were confirmed by medical records (Supplementary Table S6).

## 4. Discussion

In this large prospective cohort study of female nurses, we observed no overall association between rotating night shift work history and incident thyroid cancer. Difficulty falling or staying asleep reported most or all of the time was associated with increased thyroid cancer risk. We found evidence of joint effects of night shift work, extreme sleep duration, and sleep difficulty on thyroid cancer risk. Thus, our results provide some support for the hypothesis that co-exposure to circadian rhythm disruption and sleep disruption, i.e., shift work sleep disorder, may further increase the risk of thyroid cancer. 

Although we found no overall evidence for an increase in thyroid cancer risk with years of night shift work, our findings support potential thyroid oncogenic risks in rotating night shift workers with sleep complaints. To the best of our knowledge, no previous study has examined the association between night shift work and thyroid cancer. However, in a retrospective health surveillance study of 299 hospital employees who underwent periodic echographic examinations, rotating night shift work was associated with an increased risk of thyroid nodules, which may sometimes represent malignant lesions, compared to day work [24]. Night work has also been associated with a higher risk of benign thyroid disease, such as autoimmune hypothyroidism [25] and subclinical hypothyroidism [16,17]. Furthermore, a higher risk for thyroid diseases has been reported among physicians, an occupation with a high prevalence of night work and sleep disturbances compared to the general population [26]. 

We found evidence for independent effects of difficulty falling or staying asleep on thyroid cancer risk. This finding is consistent with results from a previous prospective cohort study showing an increased risk for thyroid cancer among postmenopausal women with higher insomnia scores [15]. In addition, we found that night workers who reported extreme sleep durations (<7 h or ≥7 h) had an increased risk of thyroid cancer. Night shift work leads to acute sleep deprivation but also chronic insomnia symptoms that may persist after quitting night shift work [27]. Therefore, disturbances in sleep duration and sleep quality in night shift workers may represent underlying mechanisms or mediators of the night shift work–cancer link. One out of four (25.6%) shift workers may develop shift work sleep disorder (SWD), which involves clinically significant insomnia symptoms, short sleep, sleepiness, and impaired daytime functioning associated with a recurrent shift work schedule [10,11]. Whether shift workers who develop SWD or other sleep disturbances are at an increased risk of chronic diseases and cancer compared to more tolerant workers (or better sleepers) is unknown. Our study suggests that maladaptation to night shift work, indicated by the report of sleep problems, might be a risk factor for thyroid cancer or a marker of increased cancer susceptibility. A similar interaction between sleep and night work exposure has been previously described for colorectal and gastric cancer [12]. Our study supports the link between the circadian clock, sleep, and thyroid cancer and shows that poor sleep might be implicated in the etiology of thyroid cancer in shift workers. 

The hypothalamus–pituitary–thyroid axis is under the control of the circadian system—with a circadian-controlled nocturnal rise in TSH levels and sleep—with a TSH release inhibition during sleep [28]. In human subjects, TSH and T3 show a strong diurnal pattern, with a clear nocturnal peak in their secretion between 2 and 4 a.m. [29]. Circadian misalignment and sleep deprivation disrupt the rhythmic TSH secretion, and TSH continues to be secreted during nocturnal sleep deprivation [30]. Therefore, morning plasma TSH levels are roughly twice as high in humans who have had a sleepless night compared to normal sleep [30]. As a result, night workers may experience higher TSH levels and an increased risk of subclinical hypothyroidism compared to their day-working counterparts, as shown in a study on female hospital workers [18]. Significantly elevated TSH and thyroid hormone levels were also observed following 24-h shifts and partial sleep deprivation [31] and in a study on sleep deprivation in young medical professionals [32]. TSH is a robust circadian marker, and its rhythm was shown to partially phase-shift in an experimental study on permanent night workers [33,34]. The elevations in TSH and thyroid hormones detected in short-term experimental sleep deprivation studies were shown to resolve quickly after recovery sleep [31]. However, our findings suggest that in the absence of sufficient or good quality recovery sleep, such changes might persist and lead to chronic thyroid dysfunction. Several studies suggest that elevated serum TSH level is linked to the incidence of thyroid cancer [19,20,35]. In sum, several lines of evidence support an association between circadian and sleep disturbances and thyroid cancer and add to the plausibility of our findings.

The main strengths of this study include the prospective cohort design, the updated shift work duration information throughout the nurses’ careers, the update of covariates throughout follow-up, and the confirmation of thyroid cancer cases using medical records. The limitations of our study should also be noted. First, rotating night shift work and sleep duration were self-assessed through a questionnaire, which may have resulted in non-differential exposure misclassification that could have contributed to some of our null findings. Second, our study evaluated the effects of the duration of night shift work exposure (cumulative years in rotating night shift schedules with at least 3 nights/month) but lacked detailed information on shift frequency (nights/month) and shift length (hours/week). However, in 2009, a subsample of nurses recalled information on their work schedule for pre-specified age ranges, and the reported number of night shifts per month was rather high and stable across the nurses’ careers, with an average of 11 night shifts/month (SD 6.0) [36]. The effects of shift work metrics such as frequency and duration and their combinations need to be considered in future studies evaluating the potential carcinogenicity of different night shift work schedules. Third, our study was conducted only among women, the majority of whom were white female nurses, and thus, our results may not apply to women with other characteristics or different shift schedules and men. Fourth, although analyses were adjusted for most of the known risk factors for thyroid cancer, multivariable models were not adjusted for radiation exposure history since this information was not routinely collected in the NHS2 cohort. If radiation exposure history is associated with shift work status, a small confounding effect could have attenuated our findings. Fifth, our analyses did not account for other potential key pathways, such as increased stress levels induced by shift work, and thus, our estimates may represent total instead of direct effects. Last, although the study was large, risk estimates in stratified analyses were unstable since these analyses were based on small numbers. 

## 5. Conclusions

In this large prospective cohort study of female nurses, we observed no overall association between rotating night shift work history and the incidence of thyroid cancer. However, our study provides evidence that frequent sleep difficulty may play a role in thyroid cancer etiology. Given the high prevalence of thyroid cancer in women and its rapidly increasing incidence, as well as the high prevalence of night shift work and disturbed sleep, the link between circadian disruption, sleep, and thyroid cancer warrants further investigation.

## Figures and Tables

**Table 1:** Age and age-adjusted characteristics according to night shift work (1989) and sleep duration categories (2001) in the Nurses’ Health Study 2 (NHS2).

	NHS2 1989 (N = 114,534)				NHS2 2001 (N = 82,786)				
	Rotating Night Shift Work History (Years)				Sleep Duration (Hours)				
	Never	1–5 Years	6–9 Years	10+ Years	5 h or Less	6 h	7 h	8 h	9 h or More
Characteristic	n = 43,524	n = 55,943	n = 9822	n = 5245	n = 4918	n = 19,879	n = 34,616	n = 18,986	n = 4387
**Age**, yrs *	34.8 (4.7)	34.5 (4.8)	35.1 (4.2)	37.3 (3.4)	47.3 (4.6)	46.9 (4.6)	46.7 (4.7)	46.5 (4.7)	46.5 (4.7)
**Race**									
White, %	96	95	95	95	91	95	97	98	97
Black, %	2	2	2	3	5	2	1	1	1
Other, %	2	3	3	3	4	3	2	2	2
**BMI**, kg per m^2^	23.9 (4.9)	24 (5.0)	24.8 (5.5)	25.3 (6.0)	28.5 (7.4)	27.4 (6.6)	26.5 (6.1)	26.4 (6.1)	27.6 (7.0)
**Physical activity**, MET-h per wk	22.7 (34.2)	25.7 (37.5)	27.4 (39.5)	32.7 (48.7)	21.9 (35.1)	21.0 (28.9)	20.5 (24.7)	21.2 (26.6)	18 (25.5)
**Smoking status**									
Never smoker, %	67	65	62	59	63	65	66	67	66
Past smoker, %	21	22	22	21	23	25	26	26	26
Current smoker, %	12	13	16	19	14	10	8	7	8
**Smoking, pack-years** ^a^	11.4 (8.2)	11.3 (8.2)	11.8 (8.2)	11.8 (8.3)	16.3 (12.9)	14.5 (11.7)	13.4 (10.7)	12.8 (10.6)	14.7 (11.8)
**Alcohol consumption**, grams per day	1.6 (0.6)	1.7 (0.6)	1.7 (0.6)	1.58 (0.6)	1.6 (0.6)	1.7 (0.6)	1.71 (0.6)	1.71 (0.7)	1.67 (0.7)
**Menopausal status**									
Premenopausal, %	98	98	98	97	71	72	75	76	74
Postmenopausal, %	2	2	2	3	29	28	25	24	26
**Postmenopausal hormone (PMH) use** ^b^									
Never, %	5	4	5	7	27	24	21	21	17
Past, %	5	8	7	7	19	15	14	15	15
Current, %	90	88	88	87	54	61	65	64	68
**Duration of PMH use**, months	19.5 (25.6)	19.1 (25.2)	20.0 (26.0)	21.2 (25.0)	45.0 (49.7)	44.8 (49.6)	42.6 (46.6)	41.2 (46.4)	46.1 (50.6)
**Ever oral contraceptive (OC) use**, %	83	83	82	83	85	86	86	86	87
**Nulliparous**, %	28	31	36	36	21	17	17	18	21
**Age at first birth** ^c^									
<25 years, %	44	39	38	46	45	41	39	39	42
25–29 years, %	43	45	44	38	40	43	45	44	42
30+ years, %	13	16	18	16	15	17	16	16	15
**Reproductive years**									
<=27 years, %	82	82	83	82	15	14	14	14	15
28–31 years, %	16	16	16	16	25	25	25	25	25
32–35 years, %	1	1	1	2	31	33	32	32	33
36–39 years, %	0	0	0	0	23	23	23	23	21
40+ years, %	0	0	0	0	6	6	6	6	5
**Breastfeeding duration ^c^**									
No breastfeeding, %	13	12	12	12	17	15	13	13	13
0.5–11 months, %	61	64	68	68	61	59	58	58	60
12–23 months, %	14	14	12	12	13	15	16	16	16
24+ months, %	11	11	8	7	9	12	13	13	11
**Hypothyroidism** history, %	5	5	5	5	19	18	18	18	23
**Hyperthyroidism** history, %	0	0	0	0	3	3	2	2	3
**Difficulty falling/staying asleep** in 2001									
Little/none of the time, %	68	68	67	62					
Sometime, %	18	18	18	19					
All or most of the time, %	14	14	15	19					
**Average hours of sleep in 24 h in 2001**									
5 h or less, %	5	6	8	11					
6 h, %	22	24	28	29					
7 h, %	42	42	39	36					
8 h, %	24	23	20	20					
9+ h, %	6	5	5	4					
**Cumulative shiftwork 1989–2001**									
Never, %					21	25	30	32	31
1–5 yrs, %					47	49	50	50	49
6–9 yrs, %					18	16	13	13	13
10+ yrs, %					14	10	7	6	7

Values are means (SD) for continuous variables, percentages or ns or both for categorical variables, and are standardized to the age distribution of the study population. Values of polytomous variables may not sum to 100% due to rounding: ^a^ Among smokers only; ^b^ Among postmenopausal women only; ^c^ Among parous women only. * Value is not age-adjusted.

**Table 2:** Rotating night shift work duration and incident thyroid cancer risk in the Nurses’ Health Study 2 (NHS2), 1989–2015.

			Age-Adjusted	Multivariable (MV) Adjusted	MV Adjusted + Sleep
	Cases (N)	Person-Yrs	HR (95%CI) ^a^	HR (95% CI) ^b^	HR (95% CI) ^c^
**NHS2 updated rotating night shift work history**					
Never	157	843,518	Ref.	Ref.	Ref.
0.1–1.9 yrs	177	785,201	1.20 (0.97, 1.49)	1.20 (0.97, 1.49)	1.20 (0.97, 1.49)
2–3.9 yrs	32	143,661	1.11 (0.76, 1.62)	1.06 (0.72, 1.56)	1.06 (0.72, 1.55)
4–5.9 yrs	116	512,508	1.21 (0.95, 1.54)	1.22 (0.95, 1.55)	1.22 (0.96, 1.55)
6–7.9 yrs	48	217,303	1.15 (0.83, 1.60)	1.17 (0.85, 1.62)	1.17 (0.84, 1.62)
8–9.9 yrs	14	72,764	0.91 (0.53, 1.58)	0.86 (0.50, 1.49)	0.86 (0.50, 1.49)
10+ yrs	44	199,332	1.10 (0.79, 1.55)	1.10 (0.78, 1.54)	1.10 (0.78, 1.54)
	588	2,774,287	*p-trend =* 0.49	*p-trend =* 0.55	*p-trend =* 0.55
**NHS2 updated rotating night shift work history**					
Never	157	843,518	Ref.	Ref.	Ref.
0.1–4.9 yrs	306	1,370,801	1.18 (0.98, 1.44)	1.19 (0.98, 1.44)	1.19 (0.98, 1.44)
5–9.9 yrs	81	360,636	1.14 (0.87, 1.50)	1.13 (0.86, 1.48)	1.13 (0.86, 1.48)
10+ yrs	44	199,332	1.10 (0.79, 1.55)	1.10 (0.79, 1.54)	1.10 (0.78, 1.54)
	588	2,774,287	*p-trend =* 0.49	*p-trend =* 0.55	*p-trend =* 0.55

^a^ Adjusted for age (months) and follow-up cycle. ^b^ Additionally adjusted for race (white, black, or other), BMI (<18.5, 18.5–24.9, 25.0–29.9, 30+ kg/m^2^), physical activity (≤8, 8.1–16, 16.1–24, >24 MET-h/week), menopausal status (premenopausal, postmenopausal), menopausal hormone therapy (never, past, current), duration of menopausal hormone therapy (continuous in months), alcohol consumption (0, 0.1–14, 14.1–28, ≥28 g/day), smoking status (never smoker, current smoker, past smoker), parity (nulliparous or parous), age at first birth (<25, 25–29, >30 yrs), reproductive years (≤27, 28–31, 32–35, 36–39, >40 yrs), breastfeeding (nulliparous, 0, 0.5–5, 6–11, >12 months). ^c^ Additionally adjusted for sleep difficulty (all/most of the time, some of the time, little/none of the time), average sleep duration in 24 h (≤5 h, 6 h, 7 h, 8 h, ≥9 h).

**Table 3:** Average sleep duration, sleeping difficulty and incident thyroid cancer risk in the Nurses’ Health Study 2 (NHS2), 2001–2015.

			Age-Adjusted	Multivariable (MV) Adjusted	MV Adjusted + Sleep
	Cases (N)	Person-Yrs	HR (95%CI) ^a^	HR (95% CI) ^b^	HR (95% CI) ^c^
**NHS2 average hours of sleep in 24 h**					
5 h or less	18	63,715	1.03 (0.63, 1.69)	1.05 (0.64, 1.73)	1.05 (0.64, 1.74)
6 h	90	261,607	1.24 (0.94, 1.62)	1.24 (0.94, 1.63)	1.24 (0.94, 1.63)
7 h	127	456,161	Ref	Ref	Ref
8 h	75	249,963	1.07 (0.81, 1.43)	1.08 (0.81, 1.43)	1.08 (0.81, 1.43)
9 h or more	20	57,168	1.21 (0.75, 1.94)	1.18 (0.74, 1.90)	1.19 (0.74, 1.91)
	330	1,088,614	*p-trend =* 0.82	*p-trend =* 0.74	*p-trend =* 0.74
**NHS2 difficulty falling or staying asleep**					
Little or none of the time	207	741,120	Ref.	Ref.	Ref.
Some of the time	66	191,573	1.26 (0.95, 1.66)	1.26 (0.95, 1.66)	1.26 (0.95, 1.66)
All or most of the time	58	151,993	1.37 (1.02, 1.83)	1.34 (1.00, 1.81)	1.34 (1.00, 1.81)
	331	1,084,685	*p-trend =* 0.02	*p-trend =* 0.03	*p-trend =* 0.03

^a^ Adjusted for age (months) and follow-up cycle. ^b^ Additionally adjusted for race (white, black or other), BMI (<18.5, 18.5–24.9, 25.0–29.9, 30+ kg/m^2^), physical activity (≤8, 8.1–16, 16.1–24, >24 MET-h/week), menopausal status (premenopausal, postmenopausal), menopausal hormone therapy (never, past, current), duration of menopausal hormone therapy (continuous in months), alcohol consumption (0, 0.1–14, 14.1–28, ≥28 g/day), smoking status (never smoker, current smoker, past smoker), parity (nulliparous or parous), age at first birth (<25, 25–29, >30 yrs), reproductive years (≤27, 28–31, 32–35, 36–39, >40 yrs), breastfeeding (nulliparous, 0, 0.5–5, 6–11, >12 months). ^c^ Additionally adjusted for cumulative nightshift work 1989–2013 (Never shift work, 0.1–4.9 yrs, 5–9.9 yrs, 10+ yrs).

**Table 4:** Rotating night shift work (updated) and thyroid cancer stratified by sleep duration and sleep difficulty (NHS2, 1989–2015).

Rotating Night Shift Work History (Yrs)	Cases (N)	HR (95%CI) ^a^	HR (95% CI) ^b^	*p*-Trend ^b^	*p*-Interaction
**ALL with sleep information**					
Never	130	Ref.	Ref.		
0.1–4.9 yrs	244	1.12 (0.91, 1.39)	1.14 (0.92, 1.41)		
5–9.9 yrs	64	1.05 (0.78, 1.42)	1.06 (0.78, 1.44)		
10+ yrs	38	1.10 (0.76, 1.58)	1.11 (0.77, 1.60)		
	476			0.69	
**<7 h of sleep**					
Never	29	Ref.	Ref.		
0.1–4.9 yrs	77	1.40 (0.91, 2.15)	1.39 (0.90, 2.14)		
5–9.9 yrs	29	1.60 (0.95, 2.69)	1.58 (0.93, 2.67)		
10+ yrs	13	1.05 (0.54, 2.03)	1.03 (0.53, 2.01)	0.56	
	148				
**7 h of sleep**					
Never	57	Ref.	Ref.		
0.1–4.9 yrs	94	1.00 (0.72, 1.40)	1.02 (0.73, 1.43)		
5–9.9 yrs	25	0.99 (0.61, 1.61)	0.95 (0.59, 1.55)		
10+ yrs	9	0.68 (0.34, 1.38)	0.66 (0.32, 1.34)	0.34	
	185				
**>7 h of sleep**					
Never	45	Ref.	Ref.		
0.1–4.9 yrs	73	1.05 (0.72, 1.53)	1.06 (0.73, 1.55)		
5–9.9 yrs	11	0.61 (0.32, 1.19)	0.63 (0.32, 1.22)		
10+ yrs	16	1.95 (1.09, 3.48)	2.17 (1.21, 3.92)	0.39	0.54
	145				
**Difficulty falling/staying asleep little or none of the time**					
Never	87	Ref.	Ref.		
0.1–4.9 yrs	158	1.08 (0.83, 1.40)	1.09 (0.84, 1.42)		
5–9.9 yrs	39	0.96 (0.66, 1.40)	0.95 (0.65, 1.40)		
10+ yrs	24	1.12 (0.71, 1.76)	1.12 (0.71, 1.77)	0.96	
	308				
**Difficulty falling/staying asleep some of the time**					
Never	29	Ref.	Ref.		
0.1–4.9 yrs	44	0.87 (0.54, 1.40)	0.90 (0.56, 1.46)		
5–9.9 yrs	14	1.05 (0.55, 2.01)	1.13 (0.59, 2.18)		
10+ yrs	7	0.72 (0.31, 1.68)	0.76 (0.32, 1.79)	0.87	
	94				
**Difficulty falling/staying asleep all or most of the time**					
Never	15	Ref.	Ref.		
0.1–4.9 yrs	42	1.86 (1.02, 3.40)	1.91 (1.04, 3.53)		
5–9.9 yrs	11	1.71 (0.77, 3.80)	1.75 (0.78, 3.95)		
10+ yrs	7	1.42 (0.57, 3.52)	1.47 (0.58, 3.73)	0.42	0.23
	75				

^a^ Adjusted for age and follow-up cycle. ^b^ Adjusted for age (months), race (white, black or other), BMI (<18.5, 18.5–24.9, 25.0–29.9, 30+ kg/m^2^), physical activity (≤8, 8.1–16, 16.1–24, >24 MET-h/week), menopausal status (premenopausal, postmenopausal), menopausal hormone therapy (never, past, current), duration of menopausal hormone therapy (continuous in months), alcohol consumption (0, 0.1–14, 14.1–28, ≥28 g/day), smoking status (never smoker, current smoker, past smoker), parity (nulliparous or parous), age at first birth (<25, 25–29, >30 yrs), reproductive years (≤27, 28–31, 32–35, 36–39, >40 yrs), breastfeeding (nulliparous, 0, 0.5–5, 6–11, ≥12 months).

## Data Availability

The data underlying this article will be shared on reasonable request to the corresponding and to the last author.

## Supplementary Materials

The following supporting information can be downloaded at: https://www.mdpi.com/article/10.3390/cancers15235673/s1, Table S1: Rotating night shift work (updated) and thyroid cancer stratified by BMI, menopausal status and chronotype (NHS2: 1989–2015); Table S2: Average sleep duration and thyroid cancer (NHS: 2001–2015), stratified by BMI, menopausal status and chronotype; Table S3: Sleep difficulty and thyroid cancer (NHS II: 2001–2015), stratified by BMI, menopausal status, chronotype and sleep duration. Table S4: Rotating night shift work duration, sleep duration and sleep difficulty and incident thyroid cancer risk restricted to papillary thyroid tumors and tumors < 2cm in the Nurses’ Health Study II, 1989–2015; Table S5: Rotating night shift work duration and incident thyroid cancer risk in the Nurses’ Health Study II, only people who answered all shiftwork questionnaires, 1989–2015; Table S6: Rotating night shift work duration and incident thyroid cancer (confirmed by medical record only) risk in the Nurses’ Health Study II, 1989–2015.

## Abbreviations

NHS2: Nurses’ Health Study 2; BMI: Body Mass Index; TSH: thyroid stimulating hormone; HR: Hazard Ratio; 95%CI: 95% confidence interval.
