# Supplementary material for: Rotating Night Shift Work, Sleep, and Thyroid Cancer Risk in the Nurses’ Health Study 2
Source: Cancers (Basel). 2023 Nov 30;15(23):5673. doi: 10.3390/cancers15235673 (PMC10705158; doi:10.3390/cancers15235673)
Supplement: Supplementary file 1 [file cancers-15-05673-s001.zip › cancers-2696546-supplementary.pdf]

Supplemental Materials

Supplemental Table S1. Rotating night shift work (updated) and thyroid cancer stratified by BMI, menopausal status and chronotype (NHS2: 1989-2015)

Rotating night shift work history (yrs)	Cases (N)	HR (95%CI) [a]	HR (95% CI) [b]	p-trend[b]	p-interaction
**BMI <30 (2001)**					
Never	121	Ref	Ref		
0.1-4.9 yrs	230	1.18 (0.95, 1.48)	1.20 (0.96, 1.49)		
5-9.9 yrs	46	0.93 (0.66, 1.31)	0.94 (0.67, 1.33)		
10+ yrs	30	1.18 (0.79, 1.77)	1.21 (0.80, 1.80)	0.86	
	427				
**BMI ≥30 (2001)**					
Never	36	Ref	Ref		
0.1-4.9 yrs	76	1.17 (0.79, 1.75)	1.20 (0.80, 1.79)		
5-9.9 yrs	35	1.54 (0.96, 2.47)	1.55 (0.97, 2.49)		
10+ yrs	14	0.90 (0.48, 1.68)	0.90 (0.48, 1.68)	0.53	0.97
	161				
**Premenopausal (2001)**					
Never	98	Ref	Ref		
0.1-4.9 yrs	188	1.11 (0.87, 1.42)	1.13 (0.88, 1.44)		
5-9.9 yrs	55	1.15 (0.83, 1.61)	1.16 (0.83, 1.62)		
10+ yrs	32	1.33 (0.89, 1.99)	1.35 (0.90, 2.02)	0.17	
	373				
**Postmenopausal (2001)**					
Never	33	Ref	Ref		
0.1-4.9 yrs	65	1.30 (0.85, 1.98)	1.35 (0.88, 2.07)		
5-9.9 yrs	16	1.21 (0.66, 2.21)	1.24 (0.67, 2.28)		
10+ yrs	6	0.57 (0.24, 1.37)	0.60 (0.25, 1.43)	0.50	0.19
	120				
**Morning persons**					
Never	82	Ref	Ref		
0.1-4.9 yrs	144	1.10 (0.83, 1.44)	1.10 (0.84, 1.45)		
5-9.9 yrs	29	0.85 (0.55, 1.30)	0.85 (0.55, 1.30)		
10+ yrs	22	1.18 (0.74, 1.90)	1.18 (0.73, 1.90)	0.91	
	277				
**Evening persons**					
Never	39	Ref	Ref		
0.1-4.9 yrs	86	1.28 (0.88, 1.88)	1.27 (0.87, 1.86)		
5-9.9 yrs	31	1.61 (1.00, 2.59)	1.55 (0.96, 2.51)		
10+ yrs	15	1.29 (0.71, 2.35)	1.21 (0.66, 2.22)	0.21	0.21
	171				

[a] Adjusted for age and follow-up cycle

[b] Adjusted for age (months), race (white, black or other), BMI (<18.5, 18.5-24.9, 25.0-29.9, 30+ kg/m2), physical activity (<=8, 8.1-16, 16.1-24, >24 MET-hrs/week), menopausal status (premenopausal, postmenopausal), menopausal hormone therapy (never, past, current), duration of menopausal hormone therapy (continuous in months), alcohol consumption (0, 0.1-14, 14.1-28, >=28g/day), smoking status (never smoker, current smoker, past smoker), parity (nulliparous or parous), age at first birth (<25, 25-29, >30 yrs), reproductive years (<=27, 28-31, 32-35, 36-39, >40 yrs), breastfeeding (nulliparous, 0, 0.5-5, 6-11, >12 months)

p-interaction: shift work*BMI: 0.83, shift work*menopausal status: 0.07, never shiftwork*morning person: 0.01, never shiftwork*evening person: 0.73, 0.1-4.9 yrs*morning person: 0.77, 0.1-4.9 yrs*evening person: 0.73, 5-9.9 yrs*morning person: 0.76, 5-9.9 yrs*evening person: 0.46, 10+ yrs*morning person: 0.22, 10+ yrs*evening person: 0.26

Supplemental Table S2. Average sleep duration and thyroid cancer (NHS: 2001-2015), stratified by BMI, menopausal status and chronotype

Sleep duration (hrs)	Cases (N)	HR (95%CI) [a]	HR (95% CI) [b]	p-trend[b]	p-interaction
**BMI <30 (2001)**					
≤6 hrs	75	1.32 (0.97, 1.80)	1.34 (0.98, 1.86)		
7 hrs	86	Ref	Ref		
8 hrs	61	1.26 (0.90, 1.75)	1.27 (0.91, 1.76)		
≥9 hrs	18	1.71 (1.02, 2.84)	1.74 (1.04, 2.89)	0.53	
	240				
**BMI >=30 (2001)**					
≤6 hrs	33	0.90 (0.57, 1.43)	0.89 (0.55, 1.42)		
7 hrs	41	Ref	Ref		
8 hrs	14	0.67 (0.36, 1.24)	0.64 (0.34, 1.18)		
≥9 hrs	2	0.30 (0.07, 1.25)	0.29 (0.07, 1.20)	0.18	0.32
	90				
**Premenopausal (2001)**					
≤6 hrs	78	1.29 (0.95, 1.75)	1.30 (0.95, 1.76)		
7 hrs	90	Ref	Ref		
8 hrs	53	1.02 (0.72, 1.43)	1.02 (0.72, 1.43)		
≥9 hrs	17	1.45 (0.86, 2.44)	1.41 (0.84, 2.37)	0.62	
	238				
**Postmenopausal (2001)**					
≤6 hrs	20	0.96 (0.53, 1.75)	0.96 (0.52, 1.77)		
7 hrs	25	Ref	Ref		
8 hrs	12	0.97 (0.48, 1.93)	0.96 (0.48, 1.93)		
≥9 hrs	2	0.64 (0.15, 2.73)	0.60 (0.14, 2.63)	0.72	0.86
	59				
**Morning persons (2009)**					
≤6 hrs	52	1.23 (0.85, 1.76))	1.22 (0.84, 1.75)		
7 hrs	70	Ref	Ref		
8 hrs	43	1.11 (0.75, 1.62)	1.11 (0.76, 1.63)		
≥9 hrs	10	1.37 (0.70, 2.66)	1.31 (0.67, 2.57)	0.97	
	175				
**Evening persons (2009)**					
≤6 hrs	36	1.25 (0.79, 1.97)	1.18 (0.74, 1.88)		
7 hrs	38	Ref	Ref		
8 hrs	22	1.13 (0.67, 1.92)	1.06 (0.62, 1.81)		
≥9 hrs	6	1.06 (0.45, 2.53)	1.02 (0.42, 2.43)	0.50	0.98
	102				

[a] Adjusted for age and follow-up cycle
[b] Adjusted for age (months), race (white, black or other), BMI (<18.5, 18.5-24.9, 25.0-29.9, 30+ kg/m2), physical activity (<=8, 8.1-16, 16.1-24, >24 MET-hrs/week), menopausal status (premenopausal, postmenopausal), menopausal hormone therapy (never, past, current), duration of menopausal hormone therapy (continuous in months), alcohol consumption (0,

0.1-14, 14.1-28, >=28g/day), smoking status (never smoker, current smoker, past smoker), parity (nulliparous or parous), age at first birth (<25, 25-29, >30 yrs), reproductive years (<=27, 28-31, 32-35, 36-39, >40 yrs), breastfeeding (nulliparous, 0, 0.5-5, 6-11, >12 months)

Supplemental Table S3. Sleep difficulty and thyroid cancer (NHS II: 2001-2015), stratified by BMI, menopausal status, chronotype and sleep duration.

Sleep difficulty	Cases (N)	HR (95%CI) [a]	HR (95% CI) [b]	p-trend[b]	p-interaction
**BMI <30 (2001)**					
Little/none of the time	152	Ref	Ref		
Some of the time	50	1.38 (1.00, 1.91)	1.40 (1.02, 1.93)		
All/most of the time	39	1.40 (0.98, 1.99)	1.43 (1.00, 2.05)	0.02	
	241				
**BMI >=30 (2001)**					
Little/none of the time	55	Ref	Ref		
Some of the time	16	0.94 (0.53, 1.65)	0.94 (0.53, 1.66)		
All/most of the time	19	1.15 (0.68, 1.94)	1.13 (0.66, 1.92)	0.98	0.06
	90				
**Premenopausal**					
Little/none of the time	154	Ref	Ref		
Some of the time	47	1.31 (0.94, 1.82)	1.28 (0.92, 1.78)		
All/most of the time	38	1.43 (1.00, 2.04)	1.36 (0.95, 1.96)	0.03	
	239				
**Postmenopausal**					
Little/none of the time	33	Ref	Ref		
Some of the time	10	1.02 (0.50, 2.09)	1.02 (0.50, 2.10)		
All/most of the time	16	1.66 (0.90, 3.05)	1.58 (0.85, 2.93)	0.41	0.74
	59				
**Morning persons**					
Little/none of the time	107	Ref	Ref		
Some of the time	37	1.43 (0.98, 2.08)	1.42 (0.98, 2.08)		
All/most of the time	32	**1.70 (1.14, 2.53)**	**1.62 (1.08, 2.42)**	**0.02**	
	176				
**Evening persons**					
Little/none of the time	67	Ref	Ref		
Some of the time	19	1.09 (0.65, 1.81)	1.05 (0.63, 1.77)		
All/most of the time	16	1.06 (0.61, 1.84)	1.00(0.57, 1.75)	0.99	0.47
	102				
**<7 hrs of sleep**					
Little/none of the time	50	Ref	Ref		
Some of the time	24	1.28 (0.78, 2.09)	1.30 (0.79, 2.12)		
All/most of the time	34	1.40 (0.90, 2.18)	1.47 (0.94, 2.30)	0.05	
	108				
**7 hrs of sleep**					
Little/none of the time	84	Ref	Ref		
Some of the time	29	1.39 (0.90, 2.12)	1.39 (0.91, 2.14)		
All/most of the time	14	1.26 (0.71, 2.22)	1.19 (0.67, 2.12)	0.31	
	127				
**>7 hrs of sleep**					

Little/none of the time	72	Ref	Ref	
Some of the time	13	0.97 (0.53, 1.76)	1.00 (0.55, 1.83)	
All/most of the time	10	1.36 (0.69, 2.65)	1.24 (0.62, 2.46)	0.91
	95			

[a] Adjusted for age and follow-up cycle

[b] Adjusted for age (months), race (white, black or other), BMI (<18.5, 18.5-24.9, 25.0-29.9, 30+ kg/m2), physical activity (<=8, 8.1-16, 16.1-24, >24 MET-hrs/week), menopausal status (premenopausal, postmenopausal), menopausal hormone therapy (never, past, current), duration of menopausal hormone therapy (continuous in months), alcohol consumption (0, 0.1-14, 14.1-28, >=28g/day), smoking status (never smoker, current smoker, past smoker), parity (nulliparous or parous), age at first birth (<25, 25-29, >30 yrs), reproductive years (<=27, 28-31, 32-35, 36-39, >40 yrs), breastfeeding (nulliparous, 0, 0.5-5, 6-11, >12 months)

Supplemental Table S4. Rotating night shift work duration, sleep duration and sleep difficulty and incident thyroid cancer risk restricted to papillary thyroid tumors and tumors <2cm in the Nurses' Health Study II, 1989-2015

	**Papillary thyroid cancer**		**Tumor size <2 cm**	
	**Cases (N)**	**HR (95%CI)[b]**	**Cases (N)**	**HR (95% CI)[b]**
**Updated rotating night shift work history**				
Never	121	Ref	97	Ref
0.1-1.9 yrs	127	1.10 (0.86, 1.42)	104	1.16 (0.88, 1.53)
2-3.9 yrs	25	1.07 (0.69, 1.66)	21	1.14 (0.71, 1.83)
4-5.9 yrs	87	1.15 (0.87, 1.52)	76	1.32 (0.98, 1.79)
6-7.9 yrs	34	1.07 (0.73, 1.57)	30	1.19 (0.79, 1.80)
8-9.9 yrs	11	0.85 (0.46, 1.59)	11	1.09 (0.58, 2.03)
10+ yrs	38	1.18 (0.81, 1.71)	35	1.41 (0.96, 2.09)
	443	*p-trend=0.55*	374	*p-trend=0.11*
**Updated rotating night shift work history**				
Never	121	Ref	97	Ref
0.1-4.9 yrs	226	1.11 (0.89, 1.39)	189	1.21 (0.94, 1.54)
5-9.9 yrs	58	1.04 (0.76, 1.43)	53	1.20 (0.86, 1.68)
10+ yrs	38	1.18 (0.81, 1.71)	35	1.42 (0.96, 2.09)
	443	*p-trend=0.55*	374	*p-trend=0.11*
**Average hours of sleep in 24 hours**				
5 hours or less	15	1.04 (0.60, 1.82)	14	1.18 (0.67, 2.09)
6 hours	73	1.23 (0.91, 1.67)	61	1.24 (0.89, 1.73)
7 hours	104	Ref	87	Ref
8 hours	62	1.10 (0.80, 1.51)	48	1.02 (0.71, 1.45)
9 hours or more	17	1.30 (0.77, 2.18)	16	1.39 (0.81, 2.38)
	271	*p-trend=0.99*	226	*p-trend=0.68*
**Difficulty falling or staying asleep**				
Little or none of the time	174	Ref	142	Ref
Sometimes	52	1.21 (0.89, 1.66)	47	1.29 (0.92, 1.80)
All or most of the time	46	1.28 (0.92, 1.78)	38	1.23 (0.85, 1.77)
	272	*p-trend=0.16*	227	*p-trend=0.20*

[b]Adjusted for age (months), race (white, black or other), BMI (<18.5, 18.5-24.9, 25.0-29.9, 30+ kg/m2), physical activity (<=8, 8.1-16, 16.1-24, >24 MET-hrs/week), menopausal status (premenopausal, postmenopausal), menopausal hormone therapy (never, past, current), duration of menopausal hormone therapy (continuous in months), alcohol consumption (0, 0.1-14, 14.1-28, >=28g/day), smoking status (never smoker, current smoker, past smoker), parity (nulliparous or parous), age at first birth (<25, 25-29, >30 yrs), reproductive years (<=27, 28-31, 32-35, 36-39, >40 yrs), breastfeeding (nulliparous, 0, 0.5-5, 6-11, >12 months)

Supplemental Table S5. Rotating night shift work duration and incident thyroid cancer risk in the Nurses' Health Study II, only people who answered all shiftwork questionnaires, 1989-2015

	Cases (N)	Person-yrs	HR (95%CI)[a]	HR (95% CI)[b]
**NHS II updated rotating night shift work history**				
Never	93	489396	Ref	Ref
0.1-1.9 yrs	96	448083	1.10 (0.82, 1.46)	1.11 (0.84, 1.48)
2-3.9 yrs	21	77737	1.21 (0.75, 1.95)	1.23 (0.76, 2.00)
4-5.9 yrs	60	288452	1.06 (0.76, 1.47)	1.08 (0.78, 1.50)
6-7.9 yrs	24	114189	1.05 (0.67, 1.64)	1.05 (0.67, 1.65)
8-9.9 yrs	9	39147	1.03 (0.52, 2.06)	1.04 (0.52, 2.07)
10+ yrs	23	99440	1.10 (0.69, 1.74)	1.10 (0.69, 1.74)
	326	1556443	*p-trend=0.74*	*p-trend=0.74*
**NHS II updated rotating night shift work history**				
Never	93	489396	Ref	Ref
0.1-4.9 yrs	167	773097	1.10 (0.85, 1.41)	1.11 (0.86, 1.44)
5-9.9 yrs	43	194510	1.05 (0.73, 1.51)	1.06 (0.73, 1.53)
10+ yrs	23	99440	1.10 (0.69, 1.73)	1.10 (0.69, 1.74)
	326	1556443		

[a]Adjusted for age (months) and follow-up cycle

[b]Adjusted for age (months), race (white, black or other), BMI (<18.5, 18.5-24.9, 25.0-29.9, 30+ kg/m2), physical activity (<=8, 8.1-16, 16.1-24, >24 MET-hrs/week), menopausal status (premenopausal, postmenopausal), menopausal hormone therapy (never, past, current), duration of menopausal hormone therapy (continuous in months), alcohol consumption (0, 0.1-14, 14.1-28, >=28g/day), smoking status (never smoker, current smoker, past smoker), parity (nulliparous or parous), age at first birth (<25, 25-29, >30 yrs), reproductive years (<=27, 28-31, 32-35, 36-39, >40 yrs), breastfeeding (nulliparous, 0, 0.5-5, 6-11, >12 months)

Supplemental Table S6. Rotating night shift work duration and incident thyroid cancer (confirmed by medical record only) risk in the Nurses' Health Study II, 1989-2015

	Cases (N)	Person-yrs	HR (95%CI)[a]	HR (95% CI)[b]
**Updated rotating night shift work history**				
Never	136	843372	Ref	Ref
0.1-1.9 yrs	139	785167	1.09 (0.86, 1.38)	1.10 (0.87, 1.39)
2-3.9 yrs	28	143606	1.13 (0.75, 1.71)	1.08 (0.72, 1.63)
4-5.9 yrs	100	512447	1.20 (0.93, 1.56)	1.23 (0.95, 1.59)
6-7.9 yrs	37	217300	1.03 (0.71, 1.48)	1.06 (0.73, 1.53)
8-9.9 yrs	13	72753	0.99 (0.56, 1.74)	0.94 (0.53, 1.66)
10+ yrs	41	199308	1.18 (0.83, 1.68)	1.20 (0.84, 1.70)
	494	2773954	*p-trend=0.45*	*p-trend=0.43*
**Updated rotating night shift work history**				
Never	136	843372	Ref	Ref
0.1-4.9 yrs	251	1370668	1.12 (0.91, 1.38)	1.13 (0.92, 1.40)
5-9.9 yrs	66	360606	1.08 (0.80, 1.45)	1.08 (0.80, 1.45)
10+ yrs	41	199308	1.18 (0.83, 1.68)	1.20 (0.84, 1.70)
	494	2773954	*p-trend=0.45*	*p-trend=0.43*
**Average hours of sleep in 24 hours**				
5 hours or less	16	63760	1.01 (0.60, 1.70)	1.06 (0.62, 1.80)
6 hours	76	261692	1.16 (0.86, 1.54)	1.17 (0.87, 1.57)
7 hours	115	456275	Ref	Ref
8 hours	68	250010	1.08 (0.80, 1.46)	1.08 (0.80, 1.46)
9 hours or more	20	57179	1.34 (0.83, 2.16)	1.33 (0.82, 2.14)
	295	1088927	*p-trend=0.73*	*p-trend=0.86*
**Difficulty falling or staying asleep**				
Little or none of the time	188	741134	Ref	Ref
Sometimes	60	191581	1.25 (0.94, 1.68)	1.25 (0.94, 1.68)
All or most of the time	48	152006	1.24 (0.90, 1.70)	1.22 (0.88, 1.68)
	296	1084721	*p-trend=0.17*	*p-trend=0.19*

[a]Adjusted for age (months) and follow-up cycle

[b]Adjusted for age (months), race (white, black or other), BMI (<18.5, 18.5-24.9, 25.0-29.9, 30+ kg/m2), physical activity (<=8, 8.1-16, 16.1-24, >24 MET-hrs/week), menopausal status (premenopausal, postmenopausal), menopausal hormone therapy (never, past, current), duration of menopausal hormone therapy (continuous in months), alcohol consumption (0, 0.1-14, 14.1-28, >=28g/day), smoking status (never smoker, current smoker, past smoker), parity (nulliparous or parous), age at first birth (<25, 25-29, >30 yrs), reproductive years (<=27, 28-31, 32-35, 36-39, >40 yrs), breastfeeding (nulliparous, 0, 0.5-5, 6-11, >12 months)
